# New Medical Institutions

**Published:** 1850-08

**Authors:** 


*New Medical Institutions.*—Two new Medical Institutions have lately been established, one in the city of New York, and one in Louisville, Kentucky.

The Faculty in the *New York Medical College* consist of Horace Green, Prof. of Medicine; Abraham L. Cox, Surgery; B. Fordyce Baker, Midwifery, &c.; John H. Whittaker, Anatomy; R. Ogden Doremus, Chemistry. The chair of Materia Medica and Pharmacy is not yet filled.

The *Kentucky School of Medicine,* as the new School at Louisville is called, opens under the direction of the following Faculty:

B. W. Dudley, M. D., Emeritus Professor of Anatomy and Surgery; Robert Peter, Chemistry, etc.; Samuel Annan, Pathology and Medicine; Josh. B. Flint, Surgery; Ethelbert L. Dudley, Anatomy; Llewellen Powell, Obstetrics; James M. Bush, Surgery; Henry M. Bullitt, Physiology and Materia Medica.

